# Nucleobases thin films deposited on nanostructured transparent conductive electrodes for optoelectronic applications

**DOI:** 10.1038/s41598-021-87181-3

**Published:** 2021-04-06

**Authors:** C. Breazu, M. Socol, N. Preda, O. Rasoga, A. Costas, G. Socol, G. Petre, A. Stanculescu

**Affiliations:** 1 National Institute of Materials Physics, 405A Atomistilor Street, P.O. Box MG-7, 077125 Magurele, Romania; 2 Plasma and Radiation Physics, National Institute for Lasers, 409 Atomistilor Street, 077125 Magurele, Romania; 3 Faculty of Physics, University of Bucharest, 405 Atomistilor Street, PO Box MG-11, 077125 Magurele, Romania

**Keywords:** Electronic devices, Nanofabrication and nanopatterning

## Abstract

Environmentally-friendly bio-organic materials have become the centre of recent developments in organic electronics, while a suitable interfacial modification is a prerequisite for future applications. In the context of researches on low cost and biodegradable resource for optoelectronics applications, the influence of a 2D nanostructured transparent conductive electrode on the morphological, structural, optical and electrical properties of nucleobases (adenine, guanine, cytosine, thymine and uracil) thin films obtained by thermal evaporation was analysed. The 2D array of nanostructures has been developed in a polymeric layer on glass substrate using a high throughput and low cost technique, UV-Nanoimprint Lithography. The indium tin oxide electrode was grown on both nanostructured and flat substrate and the properties of the heterostructures built on these two types of electrodes were analysed by comparison. We report that the organic-electrode interface modification by nano-patterning affects both the optical (transmission and emission) properties by multiple reflections on the walls of nanostructures and the electrical properties by the effect on the organic/electrode contact area and charge carrier pathway through electrodes. These results encourage the potential application of the nucleobases thin films deposited on nanostructured conductive electrode in green optoelectronic devices.

## Introduction

The use of natural or nature-inspired materials in organic electronics is a dynamic emerging research field which aims to replace the synthesized materials with natural (bio) ones in organic electronics^1–3^. Properties such as flexibility and complementary interaction with electronic structures recommend these biomaterials for integration in organic devices^4,5^. Moreover, being renewable, biodegradable and environmentally-friendly, biomaterials can lead to non-polluting technologies and effective cost reduction of the electronic devices^1,6^. In the last years, devices, such as organic field effect transistors (OFET), organic light emitting diodes (OLED), optical amplifiers, nonlinear optical electro-optical modulators fabricated with biopolymers have demonstrated high performances^7–10^.

The nucleobases (adenine, cytosine, guanine, thymine and uracil), the basic units of deoxyribonucleic (DNA) and ribonucleic (RNA) acids that constitute the genetic material for living organisms, are promising candidates as “building blocks” in nanotechnology. The molecular structures based on N-heterocyclic aromatic compounds such as pyrimidine (uracil, thymine and cytosine) and purine (adenine and guanine), determine special physico-chemical properties making them suitable for integration in electronic devices^1^.

So far, DNA and RNA were intensively investigated by biologists and chemists revealing applications of DNA in the field of OLED, OFET, photovoltaic devices, lasers, memory devices, waveguides, capacitors and solid state lightning^1^. Recent studies were focused on the use of DNA in sensors^11^, on the control of the optical properties of DNA thin solid films by doping them with highly compatible nucleobases^12^ and on a new class of materials, biopolymers, showing both the capacity for self-assembling into hierarchical structures and bioactivity, for a wide variety of organic electronic devices^13^.

But only lately the physicists became interested in exploring the physico-chemical and electronic properties of their basic units and their applications^1,5,14,15^. Nucleobases molecular engineering is a particular research field which takes into account the electronic properties of nucleic acid bases for thin-film organic devices applications, optimizing the device charge transport properties, thereby improving the device's performances^14^. One of the most important characteristic of the nucleobases that is relevant for this application field is the large range of tunability of the energy gap between the highest occupied molecular orbital (HOMO) and lowest unoccupied molecular orbital (LUMO), which favour’s the selection of the compound assuring an adequate charge transport in a specific organic device structure^2^. Compared with the DNA polymers, nucleobases are small molecules with simple structures and lower molecular weights. Due to the ability to form thin films by thermal vacuum evaporation and due to their reproducible properties, they were successfully integrated as electron blocking layers-EBL (adenine and guanine) or as hole blocking layers-HBL (cytosine, thymine and uracil) in Organic-Light-Emitting Diodes (OLEDs)^1,2,14^. Anyway, despite these significant advantages, there are only a limited number of papers about the integration of nucleobases in electronic devices^1,16^. Some analysis addressed the metal/nucleobase and inorganic semiconductor/nucleobase contact^5,17–19^, the states at these interfaces being important for the behaviour of any device.

In the context of the preparation of thin films, a special attention was paid to investigate not only the optical and conduction properties, but also the thermochemical data of the five fundamental units of the genetic code, which are known for extremely low vapour pressure and low thermal stability at elevated temperatures^20^. Recently, the interaction of nucleobases molecules with carbon nanostructures and semiconductor metal oxides and their effect on the electrical conductivity were widely studied for the development of gas sensors, biosensors and optoelectronics^21^. Taking into account the side chain engineering as a method to tailor the properties of organic compound, nucleobases represent an interesting type of functionality that may be used as an order-inducing motif^22^. Very recent studies have emphasised the possibly to create new materials with complex structure and new properties based on supramolecular self-assembled nucleobase systems using DNA technology and (bio)chemistry^23^. Other new contributions concern the synthesis of nucleobases in natural environment as a key point in the prebiotic chemical evolution^24^.

For the fabrication of any electronic device based on organic thin films, it is important to study not only the materials used as active layer(s), but also the electrodes and their interface with the active layer. In this context, the investigation of the transparent conductive electrode (TCE) plays an important role^25^, considering also its potential for applications in bioelectronics area^26^. Additionally, the fabrication of nanoscale periodic structures is a prerequisite for future applications in nanoelectronics^27^. The progress regarding the optical properties of the nano-patterned electrodes offers an interesting opportunity for developing a new class of TCE which can operate in the visible and near-infrared regime^28^. For example, by the nano-patterning process, the morphology of electrode is changed and the light inside the organic photovoltaic device (OPV) is manipulated in order to improve the efficiency^25^. Other studies report that the nanostructured electrodes used in photovoltaics can reduce the transit time between them and the active layer, leading to a reduction of the charge carriers recombination^29^. Furthermore, the light management methods in the top and bottom electrodes of organic solar cells can assure a balance between transmittance in the visible light and NIR range, which is beneficial to improve photon harvesting of active layers^30–33^. In the OLEDs case, the use of a patterned electrode could enhance the light extraction favouring the out-coupling and improving the device's external quantum efficiency^34^. Thus, the geometrical parameters of the periodic structures (nano-patterns) of the TCE are important in light harvesting and charge carriers transport^35^. Although there are many paths to fabricate nanostructured electrodes, the UV-Nanoimprint Lithography (UV-NIL) is one of the large area, mass productions technique, in which the dimensions and the uniformity of the nano-patterns can be easy controlled^25,36,37^.

Considering all these aspects, the present work aims to provide a new insight on the properties of bio-organic materials deposited on nanostructured TCEs. Thus, thin films of adenine, cytosine, guanine, thymine and uracil were deposited by thermal evaporation technique on flat and nanostructured indium tin oxide (ITO). ITO was chosen as TCE due to its unique properties as high optical transmittance, low electrical resistivity and smooth surface when deposited on glass^38^. Vacuum evaporation, the most used technique in the deposition of the organic small molecule compounds, was used with success to prepare nucleobases thin films^1,2,8,9^. The influence of the nano-patterning process on the morphological, structural, optical and electrical properties of the deposited nucleobases thin films was analysed. The results emphasize the potential application of the nucleobases thin films deposited on nano-patterned ITO in green optoelectronic devices. Till date, to our knowledge, there have not been reports on the deposition of nucleic acid bases thin films on nanostructured electrodes.

## Experimental section

### Materials

Nucleic acid bases, adenine, cytosine, guanine, thymine and uracil, in powder form were bought from Sigma-Aldrich and used without further purification. The nucleobases thin films were deposited by vacuum thermal evaporation (VTE) technique on the following substrates: nanostructured ITO/glass, ITO/glass, glass and silicon (Si).

### Preparation of heterostructures

**Figure 1:**
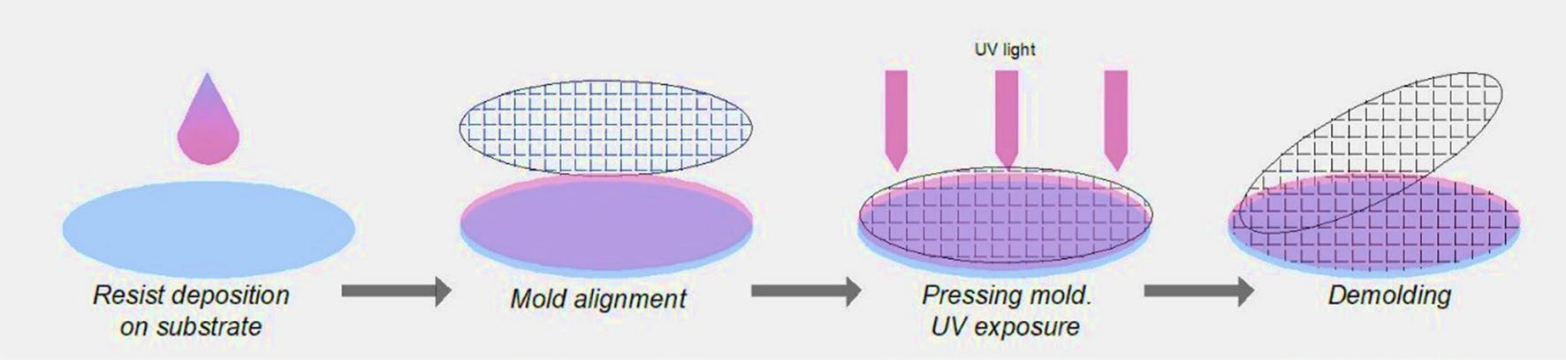
Schematic representation of UV-NIL process (P.46.0.0 AutoCad 2019, https://www.autodesk.com/products/autocad/overview?term=1-YEAR&support=null).

The nano-patterned substrates were obtained by the development, using UV-Nanoimprint Lithography (UV-NIL), of a 2D array of nano-patterns with cylindrical shape in the photoresist deposited on the glass substrates^39^. A schematic representation of the main steps involved in the UV-NIL process is shown in Fig. 1. The UV-NIL experimental configuration uses an EVG 620 mask aligner and a Brewer Science Cee 200X spin coater for thin film photoresist deposition.

Thus, cleaned glass substrates were heated at 150 °C for 120 s in order to dehydrate the surfaces for a better adhesion of the primer (EVG Prim K) and of the photoresist (EVG NIL UV/A 200 nm) thin films deposited by spinning. The deposition of each layer was followed by a thermal treatment at 120 °C for 120 s. An EVG soft stamp (negative replica of the desired nanostructures) having the following geometrical parameters ϕ = 400 nm and pitch = 1.5 μm was placed over the photoresist layer, pressed at a uniform contact pressure of 50 mbar and then exposed to UV light for facilitating its solidification. At the end of the process, the stamp is detached from the substrate leaving the desired pattern imprinted in the resist.

The transparent conductor electrode (thickness ~ 340 nm), indium thin oxide-ITO (SCI Engineered Materials Inc.) was deposited on flat and nano-patterned glass substrates by Pulsed Laser Deposition (PLD) technique using an excimer laser source with krypton fluoride (Coherent, ComplexPro 205, λ = 248 nm, FWHM = 25 ns) involving the following parameters: 1.2 J/cm^2^ for the laser fluence, 1.5 × 10^−2^ mbar for the oxygen pressure, 7000 for the number of laser pulses and 5 cm for the distance between the target and the substrate. More details regarding the ITO deposition and properties of the obtained TCE layer are given in our previous work^25^.

The deposition of the nucleobases thin films was carried out using an Alcatel system with a turbomolecular pump reaching a pressure below 2 × 10^−5^ mbar in the deposition chamber, the configuration of the evaporation system assuring a directional flux of molecules towards the deposition substrates. The metallic contact (Aluminium-Al) was also deposited by VTE on top of the organic layers at a pressure in the deposition chamber of 10^−6^ mbar with a deposition rate of 1.5 Å/s, using a K. J. Lesker “SPECTROS” system. A shadow mask was used to obtain a circular contact with an area of 12.6 mm^2^. The thickness of the Al (100 nm) was measured during the deposition with a monitoring system based on quartz crystal.

### Characterization techniques

The preservation of the chemical structure of the nucleobases during the thermal deposition was evaluated by Fourier Transform Infrared (FTIR) spectroscopy (Shimadzu IRTracer-100). The FTIR spectra of nucleobases in both forms, thin films and powders, were acquired in the 3500–500 cm^−1^ range.

The morphology of the nucleobases thin films was analysed using field emission scanning electron microscopy (FESEM) with a Zeiss Merlin Compact microscope and their structure using X-ray Diffraction (XRD) by a Bruker D8 Advance instrument operating in a Bragg–Brentano geometry with a monochromatic Cu Kα1 radiation (λ = 1.4506 Å), the diffractograms being recorded in the domain 5°-50° with a step size of 0.02° and speed of 1.5 s/step.

The optical properties of the nucleobases thin films were investigated by UV–VIS spectroscopy using a Carry 5000 spectrophotometer and by photoluminescence (PL) involving an Edinburgh Instruments spectrometer with a 450 W Xe lamp excitation and double monochromators on both excitation and emission arms. The UV–VIS spectra were acquired in the 300–800 nm range and the PL spectra were recorded at two excitation wavelengths, 335 nm and 435 nm.

The electrical measurements, I–V characteristics of the prepared structures, were performed at room temperature, in a 2-points configuration, with a Keithley 4200 SCS and a Cascade Microtech MPS 150 probe station.

## Results and discussion

### Compositional, morphological and structural properties

**Figure 2:**
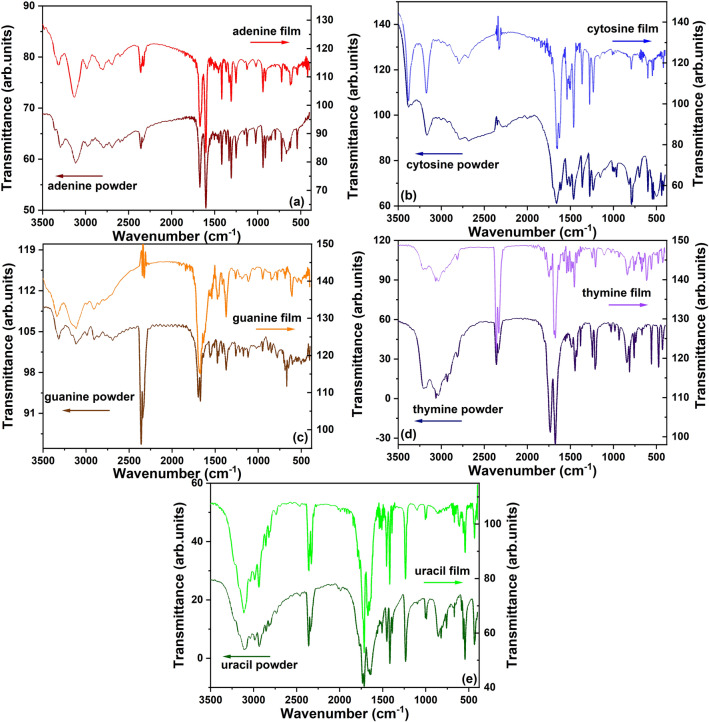
FTIR spectra of raw nucleobase powders and nucleobases thin films deposited by VTE on silicon substrates.

The FTIR spectroscopy is a powerful tool for analysing the molecular signature of the organic materials as thin films, in order to prove that their chemical structure is preserved during the deposition process. Thus, the FTIR spectra of the nucleobases thin films deposited on Si were compared with those of the raw nucleobases powders (Fig. 2), revealing a good similarity between these two spectra for each nucleobase, the characteristic vibration bands being identified in both thin films and powder forms.

For adenine film (Fig. 2a) were identified vibrations at wavenumber under 2000 cm^−1^ at about: 619 cm^−1^ (NH_2_ out-of-plane bending), 723 cm^−1^ (ring breathing), 939 cm^−1^ (C–H bending), 1309 cm^−1^ (stretching vibration of benzene), 1601 cm^−1^ (C=N stretching) and 1670 cm^−1^ (NH_2_ deformation). The higher frequencies vibrations, situated at wavenumber above 2000 cm^−1^ are characteristic to N–H and C–H bonds^40,41^. In the case of the cytosine film (Fig. 2b), were observed characteristic vibrations at about: 794 cm^−1^ (ring breathing mode), 1278 cm^−1^ and 1464 cm^−1^ (ring modes), 1543 cm^−1^ (ring stretching), 1662 cm^−1^ (C=O stretching), 3012 cm^−1^ (N–H stretching), 3171 cm^−1^ and 3383 cm^−1^ (symmetric and asymmetric N–H stretching)^42^. The guanine film (Fig. 2c) presented its specific vibrations at about: 784 cm^−1^ (CH_2_ stretching), 953 cm^−1^ and 1116 cm^−1^ (C–C stretching), 1376 cm^−1^ (C–H deformation), 1688 cm^−1^ (N–H stretching), 2905 cm^−1^ (C–H stretching), 3318 cm^−1^(OH group)^43^. For the thymine film (Fig. 2d), the FTIR characteristic features are identified at about: 667 cm^−1^ (CO_2_ bending), 847 cm^−1^ (CO wagging), 1375 cm^−1^, 1397 cm^−1^ and 1425 cm^−1^ (CH_3_ deformation), 1452 cm^−1^ (N–H in-plane bending), 1625 cm^−1^ and 1676 cm^−1^ (C=C stretching), 1741 cm^−1^ (C=O vibration), 2331 cm^−1^ and 2359 cm^−1^ (CO_2_ stretching), the doublet with peaks at 3179 cm^−1^ and 3195 cm^−1^ (N–H stretching)^44,45^. In the uracil film (Fig. 2e), the characteristic vibrations can be noted at about: 758 cm^−1^ (C=O bending), 818 cm^−1^ (C-H bending), 1004 cm^−1^ and 1232 cm^−1^ (aromatic ring), 1417 cm^−1^ (N–H stretching), 1670 cm^−1^ (C=C stretching), 1716 cm^−1^ (C=O stretching), 1737 cm^−1^ and 1771 cm^−1^ (C=O out of plane deformation coupled with C=O stretching)^44,46^.

Therefore, the FTIR results sustain that no chemical deterioration of nucleobases (adenine, cytosine, guanine, thymine and uracil) took place during their deposition by VTE, which is thus confirmed as a very adequate method for the preparation of thin films from these bio-organic compounds.

**Figure 3:**
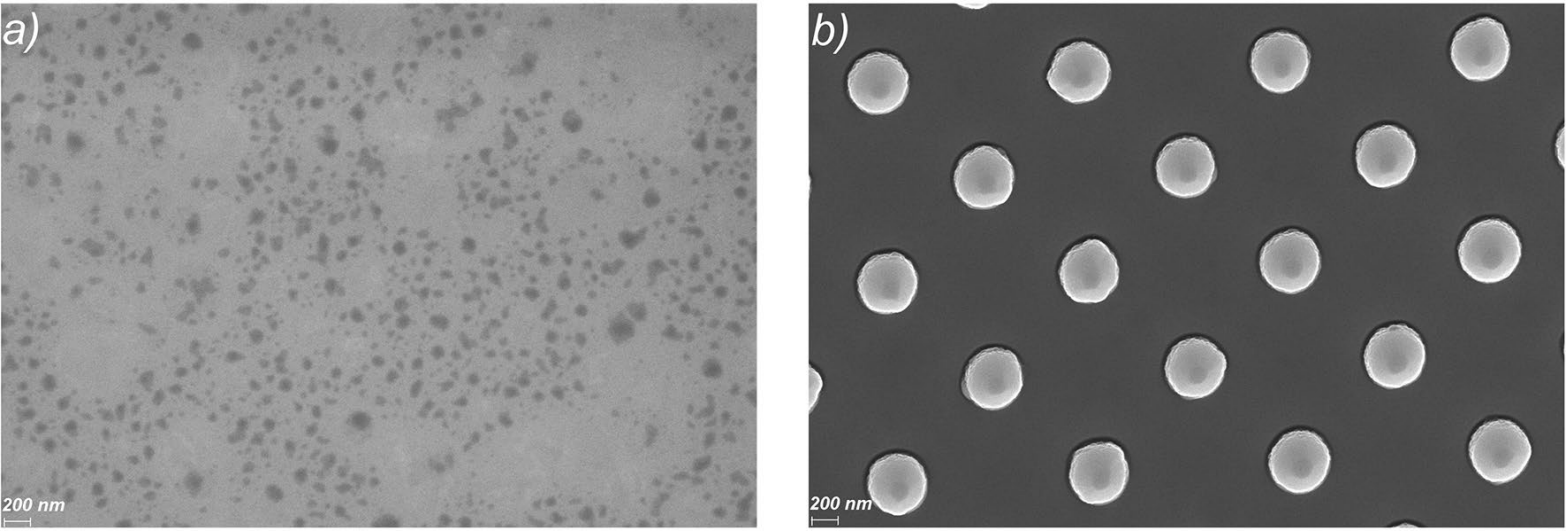
SEM images of the ITO films deposited on flat (**a**) and nano-patterned ITO (**b**).

The surface morphology of the vacuum deposited nucleobases thin films determined by the electrode surface flat or nanostructured, is important for their optical and electrical transport properties. Thus, it can be observed (Fig. 3) that the ITO layer deposited on flat glass substrate presents a smooth surface, while the one deposited on the nano-patterned glass substrate preserves the nanopillars developed in the photoresist layer. This means that there is a difference between the height of the pillars with ITO deposited on top and the height of the ITO deposited in the pitches, the space between pillars not being completely filled. Thus, the transmission of the ITO layer deposited over the periodic structures is affected by the reflectance at the interface ITO/air, the nano-patterning determining changes in the effective refractive index.

**Figure 4:**
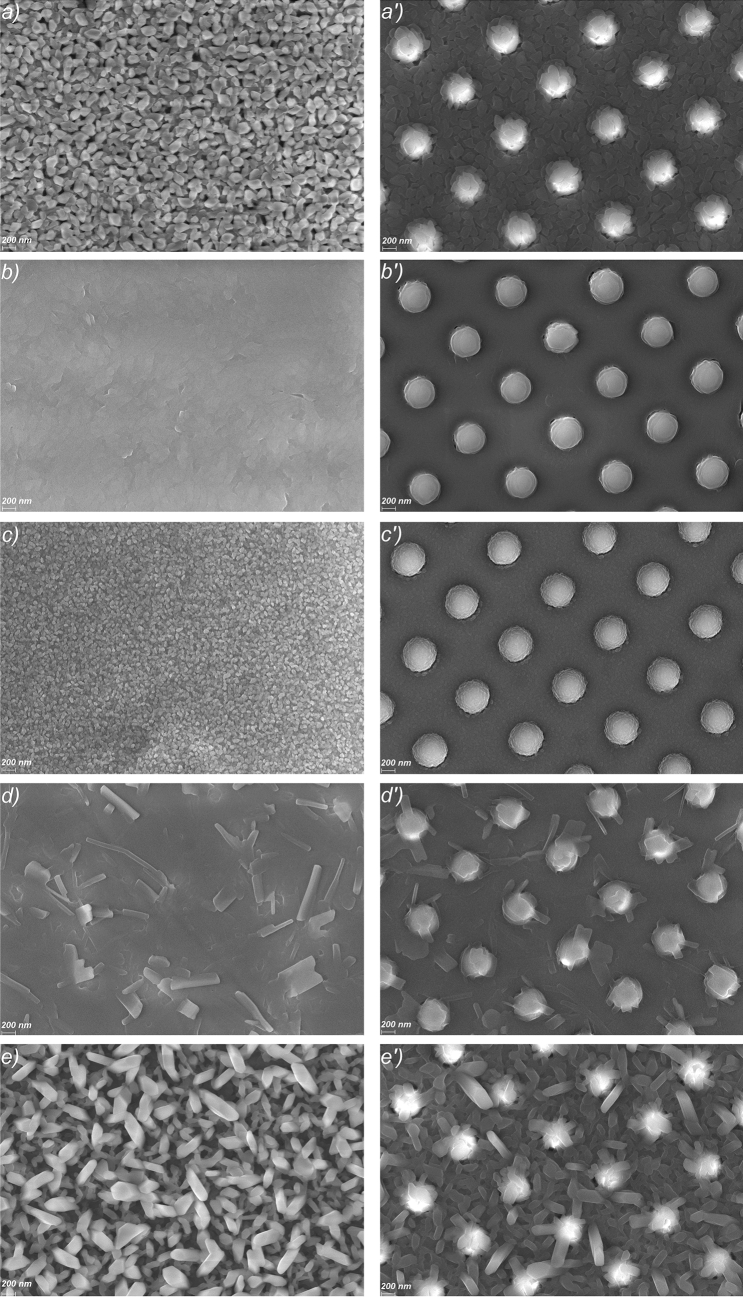
SEM images of the nucleobases, adenine, cytosine, guanine, thymine and uracil deposited on flat ITO/glass substrates (**a**–**e**) and nano-patterned ITO/glass substrates (**a'**–**e'**).

Further, the morphology of the organic thin films deposited on flat and nanostructured ITO/glass substrates (Fig. 4) were analysed. For all nucleobases, the SEM images reveal continuous layers with morphologies showing various types of grains depending on the particularities of each nucleobase (Fig. 4a–e). The films of adenine and uracil show grains with random orientation well-packed, having larger dimension and elongated shape in the case of uracil. By comparison, the film of thymine shows grains of different shape (including splinters) and dimension, randomly distributed in the layer. The presence of the nanopillars induces some changes on the morphology of the nucleobases thin films which preserve the nanostructuring. Thus, for the thymine film (Fig. 4d') the pillars present some "splinters" with different forms and orientation, while in the case of uracil film (Fig. 4e'), the nanostructured ITO is covered by clearly defined, elongated grains with varied orientation. In the adenine film (Fig. 4a') the grains are attached to the pillars forming "flowers" like structures. For adenine, thymine and uracil deposited on nano-patterned ITO the morphology is very similar with the morphology of the same layer deposited on flat ITO the only difference being the presence of nanopillars.

The cytosine film (Fig. 4b') and guanine film (Fig. 4c') deposited on nano-patterned electrode present a slight decrease in the dimension of the granulation in respect to the films deposited on flat electrode.

All these aspects can play a major role in the electrical properties of the nucleobases thin films because the morphology has a major contribution in avoiding recombination of the charge carrier and in their transport to the electrodes.

**Figure 5:**
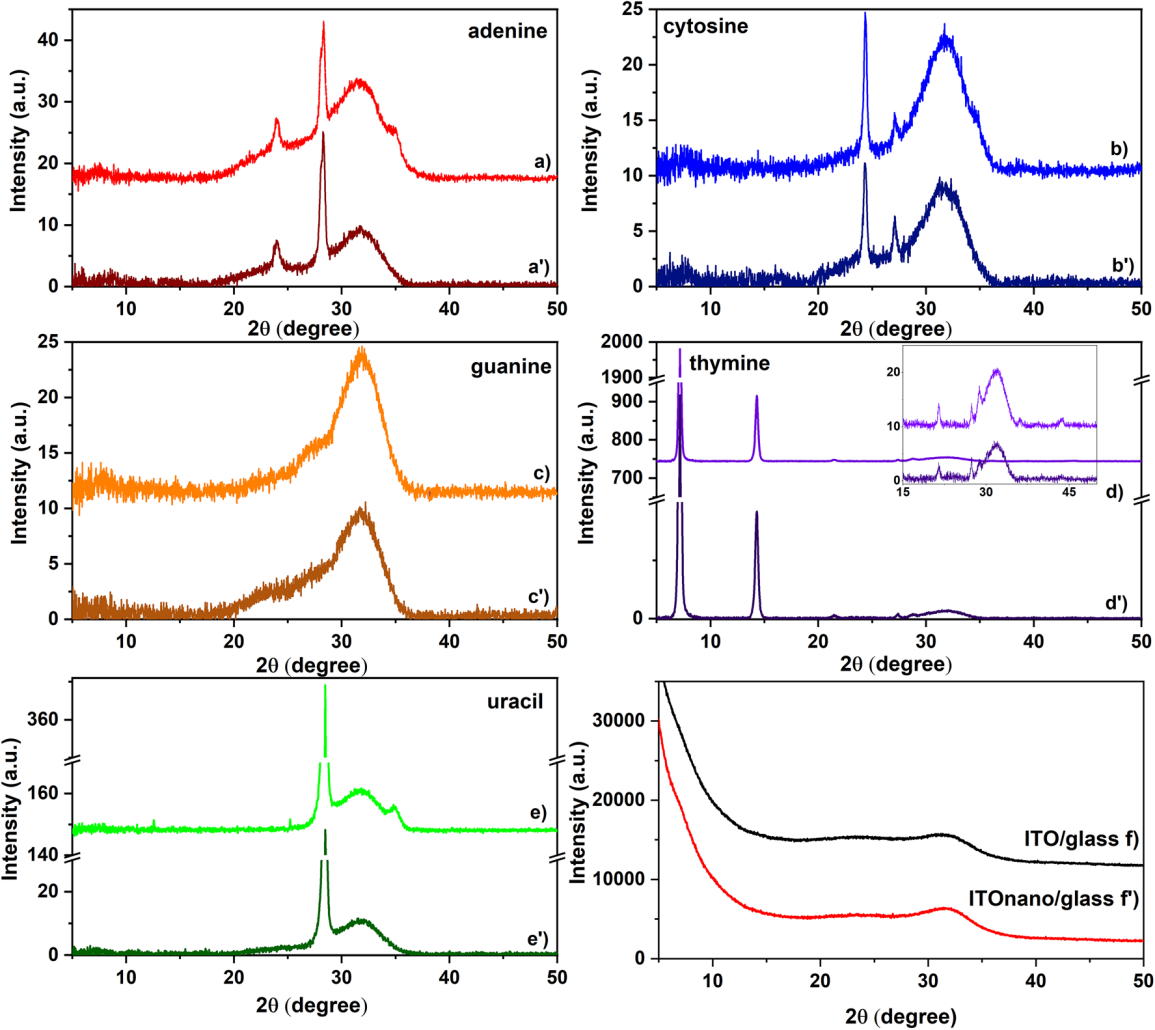
X-ray diffractograms of nucleobases thin films deposited by VTE: (**a**) adenine; (**b**) cytosine; (**c**) guanine; (**d**) thymine; (**e**) uracil on flat ITO/glass substrates and (**a'**) adenine; (**b'**); cytosine; (**c'**) guanine; (**d'**) thymine; (**e'**) uracil on patterned ITO/glass substrates; (**f**) ITO/glass substrate; (**f'**) patterned ITO/glass substrate. The inset in (**d**) shows details of the XRD pattern in the range 15°–50°.

The crystallinity is another important parameter which can influence the properties of the nucleobases thin films. The diffractograms (Fig. 5), show that the majority of the vacuum processed nucleobase thin films presents a tendency to crystallize, excepting the guanine layer. This observation is consistent with the information obtained from SEM analysis.

The XRD diagrams are almost identical for both flat and patterned deposition substrates. In the case of the adenine film (Fig. 5a), two peaks, an intense one at 29.8° and a weaker one at 24.5° (JCPDS file no. 00-004-0567) were identified. For the cytosine film (Fig. 5b), the peaks at 24.4° and 27.1° can be observed^47^. A well-defined intense peak at 28.40° is present on the both substrates coated with the uracil film (Fig. 5e). Additionally, on flat substrate, both the adenine (Fig. 5a) and uracil (Fig. 5e) films reveal another peak at 35.0°^48^. The nano-patterned substrates bring no modification in the structural properties of the cytosine, guanine and thymine film, but some small changes have been obtained for the films of adenine and uracil. The disappearance of the weak peak situated at 35.0° suggests that the nano-patterning has suppressed the development of the grains with an orientation adequate to produce reflection from the atoms planes assigned to this peak. This means that the presence of the pillars has slightly affected the crystallinity of the adenine and uracil film deposited on the patterned surface by reducing the possible orientations of the grains.

Although, the guanine film is usually obtained in crystalline state^49^, in our case, this nucleobase film is amorphous when it is deposited on both flat and patterned ITO (Fig. 5c). The deposition conditions, through the growth mechanisms, influence the properties of the deposited layer. The nucleation and growth of the film on the substrates strongly depend of the deposition parameters, such as the deposition rate, type and temperature of the substrate. Low substrate temperatures and/or low deposition rates influence the movement of the molecules on the substrate, preventing their diffusion for finding the equilibrium lattice sites and leading to an amorphous state. In our case, the most important parameter responsible for amorphous guanine thin film is the deposition rate, because the substrate is not cooled or heated. We have used a high deposition rate determined by an evaporation source temperature much higher than the evaporation temperature of guanine. Thus, the atoms had not time to move far on the substrate to find energetically favoured positions before another atom arrives at the surface and the atoms will be trapped in unfavourable positions in the growing film.

However, in the case of the thymine films, two height and sharp diffraction peaks were identified at 7.10° and 14.30° and other much lower at 21.50°; 27.40°; 28.70°; 43.60° (JCPDS file no. 00-039-1576) (Fig. 5d), suggesting a high degree of crystallinity. Thus, the XRD measurements highlighted the crystalline structure of the grains revealed on SEM images for the thin films of all nucleobases with the exception of guanine. From the XRD measurements can be concluded that the patterning process of the substrate has induced some small structural changes in the film of adenine and uracil deposited on ITOnano.

### Optical properties

**Figure 6:**
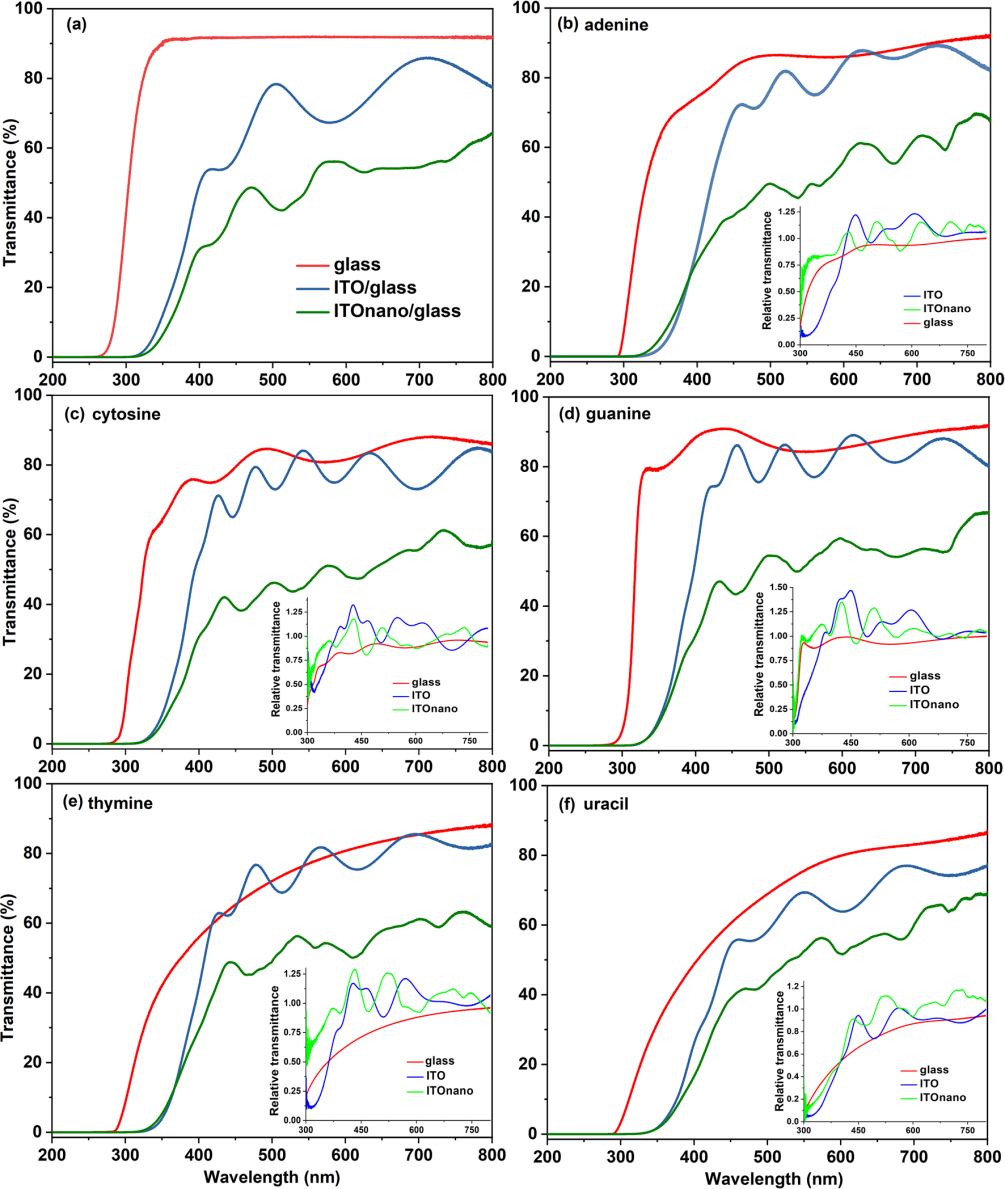
UV–Vis transmission spectra of the substrates (**a**) and of the nucleobases thin films deposited by VTE on them (**b**–**f**) with air reference. The insets represent the UV–VIS transmission spectra for the same samples with glass, ITO/glass and ITOnano/glass reference.

**Figure 7:**
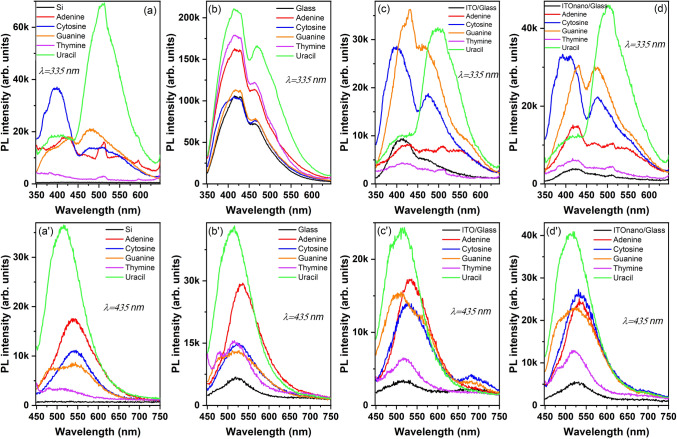
Photoluminescence spectra of nucleobases thin films deposited by VTE on silicon, glass, ITO/glass, ITOnano/glass at two excitation wavelength: 335 nm (**a**–**d**); 435 nm (**a'**–**d'**).

The optical properties of the as-prepared samples on glass, flat ITO/glass and nanostructured ITO/glass substrates were analysed by UV–VIS transmission (Fig. 6) and PL (Fig. 7) measurements. The oscillations, a succession of maxima and minima, are present in the transmittance spectrum of ITO layers (Fig. 6a) sustaining the uniformity of the film^50^. These oscillations are the result of the constructive or destructive light interference at the film interfaces with air and glass, their period being determined by the refractive index and thickness of ITO films^3^. Transmission drops are remarked on the spectrum of the ITO layer deposited on the 2D- nanostructures compared to the layer deposited on flat substrate, these bands being the result of resonant excitation of the waveguide modes supported by the transparent electrode through different diffraction order. The transmission decreases at λ ~ 400 nm and 500 nm and for wavelength longer than 560 nm because the light is trapped inside the nanostructures. The irregularities of the nano-structuring affect the shape of the absorption bands causing the bands to widen.

The pillars induce certain changes in the optical properties of the film deposited over the nanostructured ITO^28,51^ and these changes depends on the geometrical parameters of patterning (diameter and periodicity) and thickness of the film. In our case, the ITO pillars penetrated the film deposited on top of this electrode and determined the generation of nanostructures inside the nucleobase film.

From Fig. 6b–f it can be seen that the evaporated films are transparent in the visible range. The absorption peaks in the ultraviolet domain, especially under 300 nm, shown by the nucleic acid bases as a result of the π–π* transitions^43,52–54^ are hidden by the substrate that is not transparent in the UV region, under 350 nm. The adenine, guanine and cytosine films deposited on glass are characterised, due to the interference, by minima and maxima in the visible part of the spectra which confirms their uniformity. The rare interference fringes are associated with thin films. The transmission spectrum of thymine and uracil deposited on glass didn't show any interference fringes which means that they are thicker than the films of adenine, guanine and cytosine. The shape of the transmission spectra of the adenine, cytosine, guanine, thymine and uracil thin films evaporated on flat ITO/glass substrates, is dominated by the interference phenomenon in the thin layer of ITO.

The value of transmittance is high, around 80–85%, at wavelengths > 400 nm, for the films of adenine (Fig. 6b), cytosine (Fig. 6c), guanine (Fig. 6d) and thymine (Fig. 6e) deposited on ITO flat and is comparable with the transmittance of ITO flat substrate (Fig. 6a). A lower transmittance, around 60–70%, has been revealed for the uracil film deposited on ITO flat as a consequence of its thickness and/or morphological disorder (Fig. 6f). Narrow transmission resonance features have been remarked on the spectra of the nucleobase films deposited on ITO nanostructured at wavelengths > 600 nm, in the ranges 625–675 nm and 725–750 nm, confirming the increase in light absorption through light trapping inside the nanostructures.

The UV–VIS spectra of the nucleobase thin films using ITO/glass and ITOnano/glass as reference (Fig. 6b–f insets) have revealed relative transmittances > 1 which means that the transmittance of the nucleobase film is higher than that of the substrate. Thus the transmittance of the structure ITO/adenine, cytosine, guanine, thymine, uracil is higher than the transmittance of ITO because the nucleobase film acts as an anti-reflection layer. The regular reflectance (R) at the interface nucleobase/air (R_adenine-air_ = 0.074, R_cytosine-air_ = 0.017, R_guanine-air_ = 0.086, R_thymine-air_ = 0.041 and R_uracil-air_ = 0.070) is lower than the regular reflectance at the interface ITO/air, (R_ITO-air_ = 0.089), where the reflectance index, n, is: n = 1.75 for adenine^55^, n = 1.3 for cytosine^56^, n = 1.83 for guanine^57^, n = 1.49 for thymine^58^ , n = 1.72 for uracil^59^ and n = 1.85 for ITO^60^. Relative transmittance < 1 have been obtained on the nucleobases films deposited on glass. The shape of the spectra indicates a strong scattering of the thymine and uracil films for wavelengths < 600 nm.

The anti-reflecting property of the films deposited on ITOnano/glass depend on the refractive index profile determined by the nanopatterning^59,61^. The most important anti-reflecting effect has been revealed by the spectra of thymine and uracil deposited on nanostructured ITO. This behaviour could be explained by the refractive index profile determined by the surface nanostructures uniformity correlated with the thickness of the film which favour a lower reflection at the interface thymine/air and uracil/air, compared to the other nucleobases (adenine, cytosine, guanine). The absorption inside and reflections at the interfaces of the supplementary polymer layers of primer and photoresist, successively deposited on glass, involved in the UV-NIL patterning could be also be considered^17^. Additionally, the shape of the transmission resonance features is affected by the changes in the geometrical shape of the nanostructured ITO/organic interface determined by the accommodation process of the nucleobase molecules inside the cavities delimited by the ITO pillars^17^.

Thus, the nanostructures can reduce the reflection loss of the incident photons and amplify the absorption in the photoactive layer through the increase in the optical path length, offering a high potential for enhancing the cell efficiency in organic photovoltaic devices^62^*.*

The photoluminescence spectra of the nucleobases thin films were firstly recorded on layers deposited on silicon (Si) in order to eliminate the emissions which can be related to the ITO or glass substrates. In this way, an accurate evaluation of the emission properties of the nucleobases thin films can be achieved.

In Fig. 7a, can be noticed that at 335 nm excitation wavelength, except the thymine film, all other nucleobases films disclose emission bands. Thus, the adenine film reveals the characteristic weak PL band with a main maximum at ~ 420 nm^63^. The energy of the excitation wavelength, 3.70 eV, is much lower than the most recent theoretical evaluation of the optical band gap of crystalline adenine $E_{g}^{opt} = 4.39eV$, based on the dispersion corrected density functional theory (DFT) and time-dependent DFT (TDDFT)^64^.

Thus, this band with a peak at 420 nm corresponding to an energy of 2.95 eV cannot be associate to singlet (S_1_)-singlet (S_0_) transition, but a possible assignment could be to excitonic sub-band emission. After photoexcitation, a pathway to deactivation could be by the formation of a long-lived excited state with strong charge transfer character, the exciton being shared between neighbouring molecules because of the π-stacking of the nucleobase^65^. In the case of the cytosine film, two emission bands, one intense with maximum at ~ 400 nm having an un-resolved shoulder at ~ 370 nm and another broadband centred at ~ 500 nm are observed. The band with the maximum at 400 nm (3.1 eV) cannot be correlated with the singlet (S_1_)-singlet (S_0_) transition theoretically evaluated at 3.87 eV^64^. This band could be determined by sub- band excitonic de-excitation and the shoulder (3.35 eV) situated in the blue region could be determined by the deep trapped excitons de-excitation. Other study has assigned the emission peak situated at ~ 3.1–3.4 eV to singlet excimer (excited dimer) in cytosine^66^. The broad, lower intensity band in the red region (2.48 eV) could be associated to electron transitions from the triplet excited state to the ground level^67^, which is less efficient than the direct decay S_1_-S_0_. A possible explanation can be associated with the presence of an intersystem crossing (ISC) mechanism assuring the population of the triplet state^67^ , generated by the spin–orbit coupling between states (S_1_ and T_1_) of different spin multiplicity. This spin–orbit coupling together with the small gap between the singlet and triplet energy level favour’s the ISC from the S_1_ level to T_1_ level^67^. In the case of guanine, the excitation energy, 3.70 eV, can assure the transition from the ground state, the singlet (S_0_), to the first singlet excited state (S_1_), transition corresponding to an energy higher than the optical band gap theoretically evaluated at 3.61 eV^64^. In the emission spectrum of guanine has been revealed a band with maximum at ~ 430 nm (2.88 eV), that could be correlated to the sub-band excitonic de-excitation. The radiationless relaxation of the system from the excited level of S_1_ to the level of 2.88 eV could be a consequence of internal conversion. The shoulder situated in the blue region at ~ 380 nm could be assigned to the deeper exciton de-excitation and the band with maximum at ~ 480 nm to the first triplet excited state-ground state transition. Thus, the forbidden transition between triplet (T_1_) state and singlet (S_0_) state becomes allowed because of the interaction between the guanine molecules in the solid state^68^. From all nucleobases, the excitation energy of 3.70 eV is sufficient for a transition from singlet ground state S_0_ to the first excited singlet state S_1_ in uracil, the optical band gap of uracil evaluated theoretically being E_g_^opt^ = 3.7 eV^64^. The PL spectrum of the uracil thin film is characterised by two emission bands, one less intense with maximum at ~ 410 nm formed by electron transitions from the triplet T_1_ to the ground state and another with maximum at ~ 510 nm in agreement with those reported in literature^69^. The triplet state was populated by intersystem crossing (ISC) between the S_1_ and T_1_ level, this non-radiative transition becoming the reason for the appearance of phosphorescence emission at optical excitation. The peak situated at ~ 510 nm could be assigned to transitions from lower vibrational level of T_1_ to S_0_ because some molecules from the higher energy level of T_1_ (3 eV) can lose energy and pass to a lower energy level of T_1_ (2.43 eV) by a non-radiative process of vibrational relaxation. Thymine thin film showed no significant emission peaks in the spectral range 350–700 nm, despite the fact that the energy of the excitation wavelength is enough to assure the transition from the ground singlet state S_0_ to the first excited singlet state theoretically evaluate at 3.76 eV^64^.

The PL spectra of the nucleobases thin films deposited on glass substrates are dominated by the strong emission of the substrate with a maximum at ~ 420 nm and a shoulder at ~ 470 nm (Fig. 7b). ITO films deposited on both flat (Fig. 7c) and nano-patterned (Fig. 7d) glass substrates present a characteristic band of this oxide overlapped on the one linked to the glass with the maximum at ~ 410 nm^25^. Regardless the substrates, only the PL spectra of the uracil films reveal its characteristic emission band with maximum at ~ 510 nm, assigned to the de-excitation from the first triplet excited state T_1_ to ground state S_0_, as a shoulder (Fig. 7b) or as intense band (Fig. 7c,d). The shape of the PL spectra of the nucleobases films is not significantly affected by the nano-patterning of ITO. Some differences have been revealed in the intensity of the emission bands determined by the reflections inside the cavities delimited by the pillars. An apparent enhancement in the emission intensity has been shown by the films of uracil, cytosine and adenine deposited on nano-patterned ITO, probably as a consequence of the constructive reflection. It has to be mentioned that the sharp and narrow band peaked at ~ 520 nm is an artefact emission originating from the Xe flash lamp^70^.

The PL spectra obtained at 435 nm excitation wavelength, for glass substrates and ITO deposited on flat or nano-patterned glass substrates, presented in Fig. 7a', disclosed a weak large emission band with a maximum at ~ 525 nm. Thus, at the excitation with wavelength, of 2.85 eV (lower energy than the optical band gap of the nucleobases) regardless the substrate type, the emission bands attributed to the nucleobases can be easily identified: the band with maximum at ~ 510 nm for uracil films assigned to T_1_–S_0_ transition (as mentioned above) and the band with maximum at ~ 550 nm for adenine and cytosine films, probably determined also by T_1_-S_0_ transitions. The PL spectrum of the guanine film deposited on silicon substrate present a structured band with a maximum at ~ 550 nm assigned to T_1_-S_0_ transition and an un-resolved peak at ~ 480 nm which can be associated to sub-band exciton de-excitation. The PL spectrum of the nucleobase deposited on nano-patterned ITO showed an emission band with a maximum at ~ 525 nm, which is red shifted compared to emission band of the same film deposited on flat ITO (505 nm). The shoulder of the emission spectra of the film deposited on ITO flat situated at 565 nm, could be associated to un-resolved vibrational-electronic (vibronic) transition. The position of the emission peaks is affected by the arrangement of the molecules inside the cavities delimited by the pillars, the type of molecular aggregation or molecular packing, the interactions in the solid state determining a red shift because of the restrictions induced on the molecules movement. The intensity of the emission is influenced by the reflections on the walls of the cavities filled with nucleobase molecules and re-absorption of the emitted radiation.

The PL analysis emphasised that the nano-patterned substrates can induce modification in the emission properties of the nucleobases thin films through the high number of reflections on the multitude of walls of the cavities formed by the pillars of the nanostructuring^69^.

### Electrical properties

**Figure 8:**
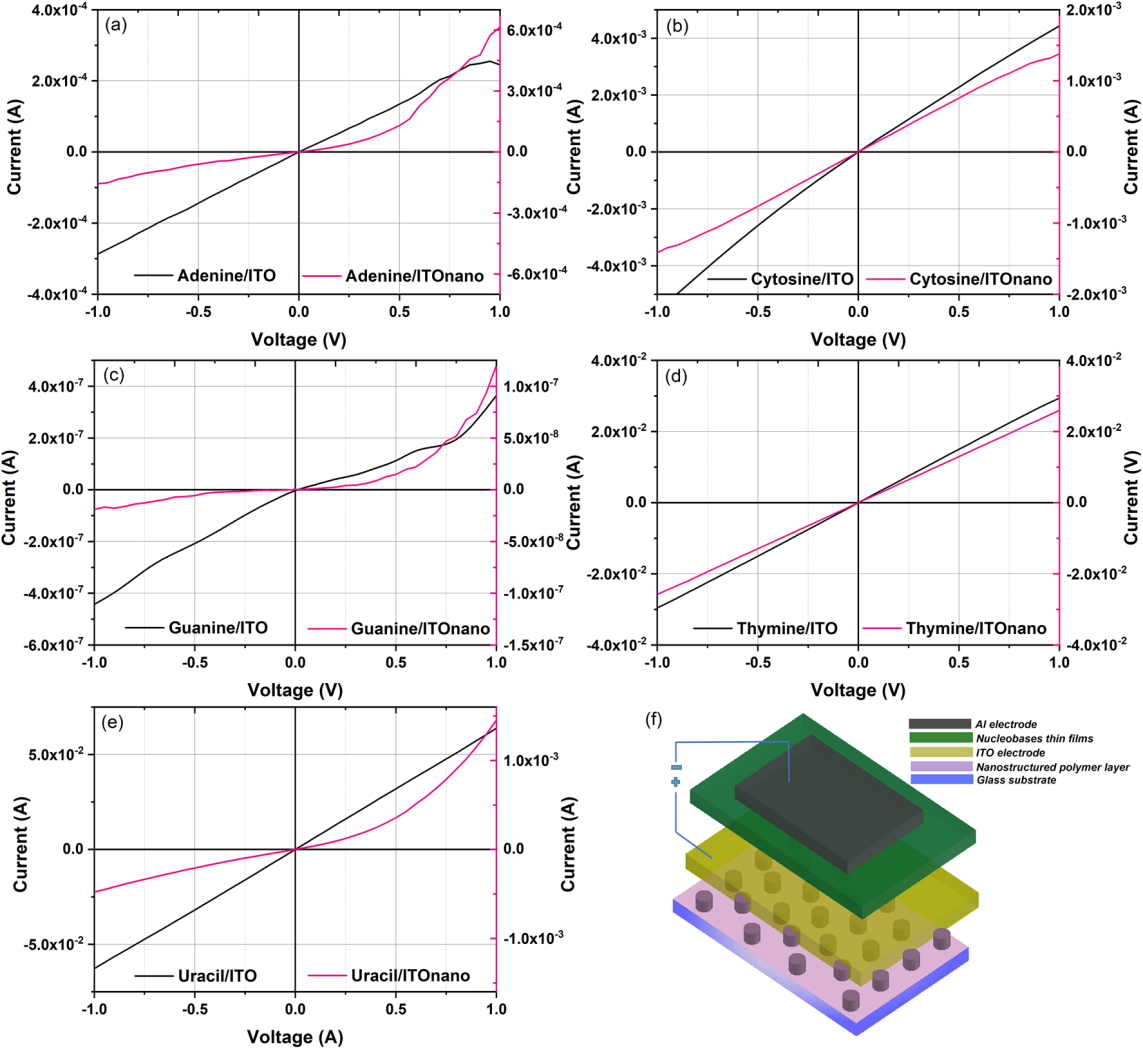
I–V characteristics of the nucleobases thin films based structures: (**a**) adenine, (**b**) cytosine, (**c**) guanine, (**d**) thymine, (**e**) uracil and (**f**) the schematic representation of the investigated heterostructures on nano-patterned TCE electrode (P.46.0.0 AutoCad 2019, https://www.autodesk.com/products/autocad/overview?term=1-YEAR&support=null).

In order to evaluate the electrical properties of the heterostructures obtained on both flat and nanostructured ITO electrode, their current–voltage (I–V) characteristics were recorded in dark conditions for an applied voltage situated between − 1 V and 1 V (Fig. 8). All the I–V characteristics plotted for evaporated nucleobases thin films on flat electrodes revealed an injection contact behaviour. A linear I–V characteristic associated to an ohmic behaviour was revealed for the heterostructures with thymine (Fig. 8d) and uracil (Fig. 8e) intermediate film and a slight deviation from linearity for the heterostructures with cytosine intermediate film (Fig. 8b). A change in the shape of I–V characteristics from linear to slightly asymmetric non-linear, is observed in the case of guanine (Fig. 8c) and adenine (Fig. 8a) films deposited on flat ITO. The interfaces ITO/nucleobase and Al/nucleobase determine the energetic barriers to be surpassed by the charge carriers. A particular behaviour is shown by adenine film (Fig. 8a) based heterostructures on flat ITO. The I–V characteristic became non-linear for applied voltage > 0.5 V because the contacts ITO/adenine and adenine/Al show different properties. At the contact with both ITO and Al adenine shows both an interface dipole and energy level bending determined by the interaction between ITO or Al atoms and bases and charge rearrangement/transfer between adenine and ITO or Al^5^. The contact ITO/thymine shows only an interface dipole, while at the contact with Al, it shows both interface dipoles and energy bending^5^. The energetic barrier for electron at the interface adenine/Al is higher than the barrier at the interface thymine/Al^5^, which means that the electrons are much easier injected from the Al electrode in thymine film. In the case of holes, the energetic barriers for both adenine and thymine are high, but smaller for adenine, which means that holes can be easier injected from ITO to adenine than from ITO to thymine. When the voltage increases the number of electrons injected from Al increases and the current increases. The difference between the ITO work function (4.70 eV) and the HOMO of adenine provides a good hole injection/transport, while the low value LUMO of adenine acts as an efficient electron blocking layer. Thus, the current is the result of the balance between the holes and electrons injected at a given voltage.

Thus, at direct polarization (ITO positively and Al negatively polarized), at an applied voltage > 0.5 eV, the energy is sufficient for initiating the hole injection from ITO, holes which might recombine with electrons attenuating the increase in the current (Fig. 8a). At reverse polarization, the minority electrons are injected from ITO electrode and finally are collected by Al electrode, their number increasing with the applied voltage. In the case of thymine, both at direct and reverse polarization the number of the charge carrier increased with the applied voltage (Fig. 8d). The hole cannot be injected in thymine from ITO positively polarized because the applied voltage is not sufficient for surpassing the energetic barrier which is higher compared to ITO/adenine contact.

The heterostructure with uracil intermediate film showed the same behaviour: an increase in the number of electrons injected from Al with the increase in the applied voltage reflected in a linear I–V characteristic and high current values (Fig. 8e).

In the heterostructure with cytosine intermediate film deposited on flat ITO (Fig. 8b), the I–V characteristic showed deviation from linearity, which are more pronounced at reverse bias. At forward bias, the barrier is too high and cannot be surpassed by holes from ITO electrode, but the current increased with voltage because the number of electrons injected from Al increased with voltage. At reverse bias the number of minority carriers injected from ITO increased more rapidly than the number of electrons injected from Al electrode at forward polarization.

At the forward polarization of the heterostructure with guanine film, the current increased slowly in the beginning and showed a sudden rise at a value of forward voltage of ~ 0.75 eV, probably because of the low resistance in forward biased condition. The current in the reverse bias is due to the flow of minority carriers, electrons, injected from ITO. With the increase in the applied voltage, the number of electrons surpassing the energy barrier at the contact ITO/guanine increased, determining an increase in current. The barrier for the electrons injection from Al to guanine is too high and cannot be surpassed applying voltages < 1 V.

The current decreases in the following order uracil > thymine > cytosine > adenine > guanine as predicted by the HOMO/LUMO levels. Uracil has the largest HOMO level from all the nucleobases and impedes the transport of holes, but its LUMO assures the lowest energetic barrier with Al from all the nucleobases, determining the most probable electron injection and transport.

The nanostructured electrode can influence the electrical properties by the modification of the electrical field with effect on the charge transport and/or collection^71^ by three different ways. Firstly, the contact area between the nano-patterned electrode and nucleobase film is larger than the area between the flat electrode and nucleobase film and the collection of the charge carrier is favoured. Secondly, the position and height of the pillars embedded in organic affect the pathway of the charge carriers to the electrodes. Thirdly, the morphology of the film characterised by grain boundaries affects the scattering/recombination processes.

A change in the shape of I–V characteristics from linear to strong asymmetric non-linear, suggesting a behaviour very close to rectifying diode, was observed in the case of adenine, guanine and uracil films deposited on nanostructured electrodes by comparison with the adenine, guanine and uracil films deposited on flat ITO (Fig. 8a,c,e).

At direct polarization the current increases when the voltage increases, slowly at the beginning and fast at voltage > 0.5 V because the number of electrons that cross the barrier increased significantly and the nanostructured shape of the ITO electrode favour’s their collection. At reverse polarization, the low current is not strongly affected by an increase in applied voltage because the number of the minority carrier crossing the barrier and injected from the ITO electrode for voltages < 1 V is reduced. Additionally, the nanostructuring of ITO determines longer pathway for a part of these electrons which favour’s their recombination on trapping centres and/or scattering on grain boundaries.

The I–V characteristics for the heterostructures with thymine and cytosine film are not significantly affected by the nano-patterning of the ITO electrode. They preserved the symmetric linear (Fig. 8d) or slightly symmetric non-linear (Fig. 8b) injector contact behaviour.

For the thymine films, the ohmic behaviour with similar high values of the current, 2.9 × 10^−2^ A in the case of the film deposited on flat ITO and 2.6 × 10^−2^ A for the film deposited on nanostructured ITO (for an applied voltage of 1 V), the large grain morphology (Fig. 4d) correlated with higher degree of crystallinity (Fig. 5d) contributing together with other mentioned factors to this favourable result. Thymine films were previously used as EBL in OLED structures, because improves the outcoupling efficiency of the device due to the emitted light dispersion occurring in the microcavities presented by the organic film^2^. Thus, using a nano-patterned substrate can be improved the efficiency of the electronic device.

For the guanine films, lower current values, 3.6 × 10^−7^ A and 1.2 × 10^−7^ A (with more than three order of magnitude compared to the other nucleobases) at an applied voltage of 1 V were recorded. However, in our case, the morphology of the guanine film (Fig. 4c') seems to be an important factor affecting the transport and collection of the carriers at the electrodes.

By nanostructuring of the ITO electrode, the behavior of the heterostructure realized with adenine, guanine and uracil nucleobase intermediate film can be changed from linear to rectifying. An increase in the current by nanostructuring has been obtained only for the heterostructure with adenine film. All the other heterostructures realized on nanostructured ITO has shown a lower current than the same heterostructure realized in flat ITO. This is the result of the scattering and recombination processes taking place at the grain boundaries on the pathway to the electrodes of the charge carriers. The morphology determines the number of grains boundaries involved in the charge carrier scattering. The pathway is affected by the geometrical parameters of the nano-patterning and thickness of the film determining the penetration depth of the ITO pillars into the organic film. Thus, the advantages of the electrode nanostructuring and increased area for the charge carrier collection can be significantly diminished, resulting a lower current, as a consequence of the geometrical parameters of nano-patterning, (e.g. height of pillars), film thickness and morphology of the deposited material. The small structural modifications shown by the adenine and uracil films deposited on ITOnano can not be associated with the changes from linear to non-linear asymmetric of I–V characteristics.

Consequently, nanostructured transparent electrode can induce modifications in the optical and electrical properties of the nucleobases thin films, the results of this study being useful in the field of organic devices, especially as buffer layers for OLEDs.

## Conclusions

A comparative study between the properties of the nucleic acid bases thin films prepared by VTE technique on ITO deposited on flat and nano-patterned glass substrates was carried out. The 2D array of nanostructures were successfully fabricated using UV-Nanoimprint Lithography technique. Further, ITO layers were deposited as transparent conductive electrode on flat and nano-patterned glass by PLD technique. The organic layers of adenine, cytosine, guanine, thymine and uracil evaporated on flat and nanostructured electrodes were investigated from structural, morphological, optical and electrical point of view.

The preservation of the characteristic vibrations of the chromophoric group from the nucleobases molecules during the VTE deposition confirms the suitability of this method for preparing these materials as thin films. SEM imagines evidenced the presence of the 2D array of nanostructures after the deposition of ITO electrode and the nucleobases thin films and also the molecular arrangement of the organic layers depending of the particularities of each base. X-ray diffraction shows preferential orientation of the organic films. The nano-patterning of the substrate has induced some modifications in the crystallinity of adenine and uracil films. Compared to the films obtained on flat electrode, those deposited on nano-patterned electrode evidence an increase in absorption due to the decrease of the incident photons loss by multiple reflections. The emission properties of the organic films deposited on nanostructure electrodes are affected by the reflections on the walls of the cavities, filled with nucleobase molecules, delimited by the pillars of the nanostructure and re-absorption of the emitted radiation. The I–V characteristics recorded in dark conditions have shown an ohmic contact behaviour for the heterostructures with cytosine and thymine films obtained on both flat and nanostructured ITO electrodes. Exceptions are the heterostructures prepared with adenine, guanine and uracil film on nano-patterned substrates showing a behaviour close to rectifying diode as a consequence of energetic criteria, large collection area of the electrode, pathway of the charge carriers and morphology of the film. The best current values were achieved for the films of nucleobases characterized by higher value LUMO (uracil, thymine and cytosine), which also show similar HOMO–LUMO gap.

The nano-patterned transparent conductive electrode induced changes in the optical and electrical properties of the nucleobases thin films deposited on it. As consequence, the nanostructured films of nucleobases are promising candidates for applications in the electronic and optoelectronic area as electron or hole blocking layer depending on the value of the higher occupied molecular orbital (HOMO) energy.
